# 

**Published:** 1906-07-21

**Authors:** 


### CONTENTS.

**The Compulsory Notification of Tuberculosis** - 275
**An Appeal for the Child** - 276
**Annotations** - 277
**Medical Opinion and Movement** - 278
**Graduate Teaching Facilities in London Hospitals** - 279
**Remedies and their Uses** - 280
**Practical Notes on Diagnosis and Treatment** - 284
**Hospital Clinics—**
Irregularities of the Heart - 281
Cerebro-Spinal Meningitis in Great Britain - 283
**Special Analytical and Health Commission on Tinned and Preserved Food** - 285
**Hospital Administration—**
Current Hospital Topics - 287
Opening of the Royal Victoria Infirmary, Newcastle - 288
Social and Poor-law Problems - 289

*NURSING SECTION.*

**Notes on News from the Nursing World—**
The Education Bill and Queen's Nurses—Nursing the Injured at Handcross—Economy at the Expense of Efficiency—Disobedient and Incapable Midwives—Overwork at Aston—A Movement to Lower the Standard—Imperial Military Nursing Service—The Late Mr. Edward Rawlings—The Indian Nursing Association—Garden Fête for a Bed at Guy's Hospital—Interesting Ceremony at St. Mary's Hospital—Sussex County Nursing Association—A Successful Pound Day—Bellevue Hospital and the Training School—Short Items - 225-228
**The Nursing Outlook** - 229
**The Care and Nursing of the Insane** - 230
**The Nurse's Clinic** - 231
**Incidents in a Nurse's Life** - 232
**The Victorian Order of Nurses in Vancouver** - 232
**Central Midwives Board** - 233
**General Lying-in Hospital** - 234
**Presentations** - 234
**Everybody's Opinion** - 234
**Appointments** - 236
**A Book and Its Story** - 237
**Notes and Queries** - 238
**For Reading to the Sick** - 238

